# Bactericidal mechanisms and effector targets of TiO__2__ and Ag-TiO__2__ against *Staphylococcus aureus*

**DOI:** 10.1099/jmm.0.000457

**Published:** 2017-04-28

**Authors:** Xuhong Jiang, Bin Lv, Yuan Wang, Qianhong Shen, Xinmin Wang

**Affiliations:** ^1^​ Department of Emergency, the First Affiliated Hospital of Zhejiang Chinese Medical University, Hangzhou, PR China; ^2^​ Department of Gastroenterology, the First Affiliated Hospital of Zhejiang Chinese Medical University, Hangzhou, PR China; ^3^​ Zhejiang-California International NanoSystems Institute, Zhejiang University, Hangzhou, PR China

**Keywords:** TiO__2__, Ag^+^, *Staphylococcusaureus*, bactericidal mechanism, effector targets

## Abstract

**Purpose:**

In our previous study, Ag^+^-loaded TiO_2_ and Ag^+^-loaded SiO_2_ coatings for tracheal intubation were prepared to prevent ventilator-associated pneumonia (VAP), but the antimicrobial targets and the underlying mechanisms of TiO_2_ and Ag-TiO_2_ (Ag^+^) are still unclear. We attempted to elucidate the antimicrobial activity and potential mechanisms against *Staphylococcus aureus*.

**Methodology:**

The study tested the TiO_2_ and Ag^+^ bacteriostatic activity against *S. aureus* strains by MIC assays and *S. aureus* growth curves, lesion in the membranes by surface hydrophobicity tests, conductivity tests and measurements of DNA and RNA contents in *S. aureus* cultures, and investigated the inhibition of soluble protein and nucleic acid synthesis by measurements of soluble protein content, fluorescent intensity and nucleic acid content of living *S. aureus*.

**Results:**

The MIC values of TiO_2_ and Ag^+^ were 1.6 mg ml^−1^ and 5.781 µg ml^−1^. TiO_2_ and Ag^+^ could inhibit the growth of *S. aureus*. After treatment with TiO_2_ and Ag^+^, the surface hydrophobicity was significantly reduced, the conductivity of cultures increased, and DNA and RNA content in cultures showed no obvious changes. The expressions of soluble proteins and nucleic acid contents of living *S. aureus* were reduced after treatment with TiO_2_ and Ag^+^.

**Conclusion:**

TiO_2_ and Ag^+^ could cause slight lesion in the membrane to affect *S. aureus* membrane permeability, but not decomposition of membrane. Moreover, TiO_2_ and Ag^+^ could lead to reduced expression of soluble protein by inhibiting the synthesis of nucleic acids, thereby further inhibiting the growth of *S. aureus*.

## Introduction

TiO_2_ is one of the most important semiconducting oxides because of its photocatalytic activity, conservative properties, low cost, low toxicity and stability in response to light illumination [1] However, TiO_2_ has a high band gap energy (3.2 eV), which limits its wide applications in the visible light range of the solar spectrum [2, 3]. Additionally, the generation of reactive oxygen species by TiO_2_ is also inhibited by its low electron transfer rate to oxygen and high rate of electron–hole recombination [4]. To enhance the generation of reactive oxygen species by TiO_2_ nanomaterials under conditions of visible light irradiation, noble metals such as Pd, Pt, Au and Ag have been used to modify TiO_2_ [5–11].

Among these noble metals, Ag has attracted significant attention for doping TiO_2_ to inactivate bacteria because of its excellent broad-spectrum antibacterial properties, heat resistance, good dispersion and low drug resistance [12].

We prepared antimicrobial films that contained Ag-TiO_2_ and Ag-SiO_2_ polyethylene endotracheal tubes that had excellent antimicrobial properties against ventilator-associated pneumonia (VAP) [13]. Zhou *et al.* found that Ag^+^-TiO_2_ nanobelts exhibited greater photocatalytic degradation activity against methyl orange than TiO_2_ because of the greater amounts of reactive oxygen species that are generated [14]. Because the inactivation of bacterial cells shows a correlation with the photocatalytic decomposition of organic matter, nanobelts with Ag^+^-deposited TiO_2_ are expected to have improved bactericidal properties under conditions of visible light irradiation [15]. Recently, the bactericidal mechanism and biosafety of Ag^+^ was investigated by researchers. Heidenau *et al.* found that an Ag^+^ concentration of 3.5 µM was not cytotoxic to mouse L929 fibroblasts [16]. Baldi *et al.* found that an Ag^+^ concentration between 30 and 70 µM affected both cell viability and morphology [17]. Jin *et al.* found that, for both Gram-negative *Escherichia coli* ATCC 15597 and Gram-positive *Bacillus subtilis*, the antimicrobial effects of Ag^+^ were greater under light conditions compared with those under dark conditions [18]. Tan *et al.* found that Ag nanoparticles exhibited efficient microbicidal activity against *Staphylococcus aureus*, but did not significantly affect the viability of osteoblast cells (MC3T3-E1) [19]. Shi *et al.* found that, compared with TiO_2_-SiO_2_, Ag-TiO_2_-SiO_2_ exhibited higher photocatalytic activity and antibacterial efficiency after uniform Ag nanoparticles were loaded onto TiO_2_ [20]. In response to an Ag concentration of 0.6 mg ml^−1^, the antibacterial rates against *Escherichia coli* and *S. aureus* were 93.41 and 97.37 %, respectively. Jin *et al.* proposed that the potential antibacterial mechanisms of Ag^+^ were as follows: first, Ag^+^ can be easily adsorbed onto the surface of *E. coli* and affect the outer membrane permeability and ﬂuidity; second, Ag^+^(silver nanoparticles with carbon dots doped with sulfur) with a smaller size (7.3±1.0 and 6.1±0.8 nm) can permeate the membrane of *E. coli* to interact with DNA and components of the respiratory chain; and third, Ag^+^ release can cause *E. coli* death [21]. However, as Ag nanoparticles are ionic antibacterial implant materials, the best way to achieve good bactericidal or anti-adhesion properties of medical devices without harming normal tissues or cells remains a common subject of debate.

The preparation and theoretical use of TiO_2_ and Ag^+^ antibacterial agents have been studied by our research group for many years [11, 22]. In this present study, we aimed to fabricate TiO_2_ and Ag^+^ antibacterial agents and investigate their bactericidal mechanisms. We attempted to elucidate the antimicrobial activity and potential mechanisms of TiO_2_ and Ag^+^ as antibacterial agents against *S. aureus* by determining their antimicrobial activity of the MIC values and *S. aureus* growth curve, conductivity, and macromolecular substances in culture medium, and by measuring the soluble proteins and nucleic acids of *S. aureus* after treatment with antibacterial agents.

## Methods

### Reagents and materials

2,3,5-triphenyltetrazolium chloride (TTC), hexadecane, 4′,6-diamidino-2-phenylindole (DAPI), hexadecane, protein marker and distilled water were of guaranteed reagent quality. The TiO_2_ stock solution (aged 60 days) was prepared according to previously published methods [22]. Ag^+^ stock solution was prepared so that the Ag-TiO_2_ mesoporous materials were dispersed into the water at a mass concentration of 5 wt% [11]. *S. aureus* ATCC 25923 was obtained from Hangzhou 100 Biotechnology. The biosafety cabinet and enzyme marker were produced by Thermo. The fluorescence microscope was produced by Olympus. Vertical electrophoresis apparatus was produced by Tanon. The conductivity meter was produced by Shanghai Dapu Instrument Co. The UV spectrophotometer was produced by Shanghai Xinmao Instrument Co. The decolorization table was produced by Shanghai Huxi Instrument Co.

### Reagent preparation

#### Broth medium

Beef extract (3 g), peptone (10 g) and NaCl (5 g). All salts were dissolved in 800 ml distilled water, the pH was adjusted to 7.2–7.4, and then more distilled water was added to achieve a total volume of 1000 ml.

#### PBS buffer

PBS buffer contained Na_2_HPO_4_ (14.6 g), NaH_2_PO_4_ (1.48 g) and NaCl (43.83 g). All salts were dissolved in 1 l distilled water, and water was added to achieve a final volume of 5 l. The pH was adjusted to 7.2–7.4 with NaOH and stored at room temperature after autoclave sterilization.

### Experimental groups

(1) Control; (2) TiO_2_ groups: 0.1, 0.2, 0.4, 0.8 or 1.6 mg ml^−1^; (3) Ag^+^ groups: 0.723, 1.445, 2.891, 5.781 or 11.560 µg ml^−1^.

### MIC assay

A few *S. aureus* were picked up from agar/slant culture medium and inoculated in broth medium at 37 °C, 120 r.p.m. for 24 h. The final concentration of cells was adjusted to 10^6^–10^7^ c.f.u. ml^−1^ with broth medium. Then, 1.5 ml of the bacterial culture was inoculated on 12-well plates in which 10 µl serial dilutions of TiO_2_ or Ag^+^ were added. Plates were incubated at 37 °C, 120 r.p.m. for 24 h. Subsequently, 150 µl of 0.2 % TTC was added to each well. Changes in colour were observed after incubation at 37 °C, 120 r.p.m. for 4 h in the dark. Each group included triple technical replicates.

### *S. aureus* growth curves

The *S. aureus* cells were incubated to the log-phase of growth. Next, 2 % inoculum was transferred to fresh broth medium with 0.5 MIC TiO_2_ or Ag^+^ and incubated at 37 °C, 120 r.p.m. Samples were obtained and the OD value was determined at 580 nm at 4 h intervals. Each group included triple technical replicates. *S. aureus* growth curves after TiO_2_ or Ag^+^ treatment were plotted as OD values on the vertical axis compared with time on the horizontal axis. Each group included triple technical replicates.

### Surface hydrophobicity of *S. aureus*

*S. aureus* cells were incubated until they reached the log-phase of growth. Then, 2 % inoculum was transferred to fresh broth medium with 0.5 MIC TiO_2_ or Ag^+^ and incubated at 37 °C, 120 r.p.m. Next, 2 ml samples were transferred into 5 ml tubes and, after incubation for 0, 2, 8 or 16 h, they were centrifuged at 5000 r.p.m. (Allegra X-15R; Bechman) for 10 min. Cell pellets were washed twice with 0.1 M KNO_3_ (pH 6.2) and resuspended with the same buffer. Cell suspensions were adjusted to absorbance *A*_400_=0.4 (i.e. *A*_0_=0.4); 10 mM sodium phosphate buffer solution served as a blank control. Additionally, 0.2 ml hexadecane was added into the adjusted cell suspensions. Samples were mixed for 2 min using a vortex after a 10 min static incubation. Aqueous phases were transferred and the *A*_400_ (*A*_1_) absorbance values were measured until the two phases of the mixture became completely separated. Bacterial surface hydrophobicity was indicated by the absorption rate. Bacterial absorption rate =(1−*A*_1_/*A*_0_)×100 %. Each group included triple technical replicates.

### Conductivity tests of *S. aureus* cultures

*S. aureus* cells were incubated until they reached the log-phase of growth. Next, 2 % inoculum was transferred to fresh broth medium with 0.5 MIC TiO_2_ or Ag^+^ and then incubated at 37 °C, 120 r.p.m. Thereafter, 5 ml samples were harvested after incubation for 0, 2, 4, 6 and 8 h, which were each centrifuged at 4000 r.p.m. for 10 min. Cell conductivity was determined with a conductivity meter after 20-fold dilutions of supernatants. Each group included triple technical replicates.

### Measurements of DNA and RNA contents in cultures of *S. aureus*

*S. aureus* cells were incubated until they reached the log-phase of growth. Then, 2 % of the inoculum was transferred into fresh broth medium and was again incubated until the culture reached log-phase at 37 °C, 120 r.p.m. Cells were collected, washed twice with PBS and resuspended at an appropriate concentration to yield a cell suspension. A final concentration of 0.5 MIC TiO_2_ or Ag^+^ was added to the prepared suspension. After treatment for 0, 2, 4, 6 or 8 h, 3 ml of the suspension was collected and centrifuged at 4000 r.p.m. (Allegra X-15R; Bechman) for 10 min. Absorption values of DNA and RNA in the supernatant were measured at 260 nm using an ultraviolet spectrophotometer. Each group included triple technical replicates.

### Measurement of soluble protein content in *S. aureus*

*S. aureus* cells were incubated until they reached the log-phase of growth. Then, 2 % of the inoculum was transferred into fresh broth medium with 0.5 MIC TiO_2_ or Ag^+^ and incubated at 37 °C, 120 r.p.m. Suspensions were harvested after incubation for 12 and 16 h, and 0.1 g cells were centrifuged at 4000 r.p.m. (Allegra X-15R; Bechman) for 10 min. Pellets were collected and resuspended in 40 µl sterile water. The sample buffer was mixed with the sample at a volume ratio of 1 : 4, and the mixture was heated in boiling water for 7 min and then centrifuged at 4000 r.p.m. (Allegra X-15R; Bechman) for 10 min. Soluble proteins in the supernatant were assessed by SDS-PAGE. Each group included triple technical replicates.

### Fluorescence intensity measurements of *S. aureus*

*S. aureus* cells were incubated until they reached the log-phase of growth. Then, 2 % of the inoculum was transferred into the fresh broth medium with 0.5 MIC TiO_2_ or Ag^+^ and was incubated at 37 °C, 120 r.p.m. for 24 h. Aliquots of 10 µl per sample were diluted 200-fold. Subsequently, 100 µl diluted suspension was spotted on a microslide and stained with the same volume of DAPI staining solution. Mixtures were incubated without shaking in the dark for 10 min. Fluorescence intensity was measured using a fluorescence microplate reader and imaged. Each group included triple technical replicates.

### Nucleic acid content measurements of *S. aureus*

*S. aureus* cells were incubated until they reached the log-phase of growth. Then, 2 % of the inoculum was transferred into the fresh broth medium with0.5 MIC TiO_2_ or Ag^+^ and was incubated at 37 °C, 120 r.p.m. Then, 50 µl aliquots were harvested after incubation for 12, 16, 20 and 24 h, and stained with 150 µl DAPI staining solution. Prepared samples were measured immediately after shaking for 10 min. The fluorescence parameters were 364 nm to measure the DNA content and 400 nm to measure the RNA content. Each group included triple technical replicates.

### Statistical analysis

All data are presented as mean±sd. Differences between group means were assessed by ANOVA for multiple comparisons using spss 16.0. Values of *P*<0.05 were considered significant.

## RESULTS

### MIC values of TiO_2_ against *S. aureus*

**Fig. 1.**
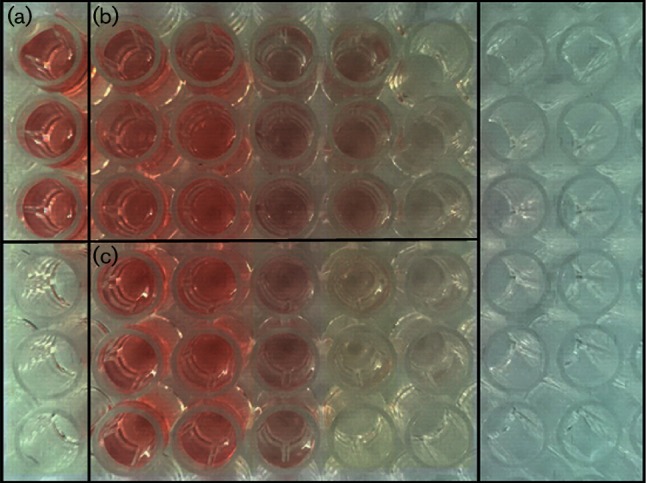
Determination of MIC values for TiO_2_ and Ag^+^ against *S. aureus*. (a) Control. (b) Groups treated with TiO_2_:0.1, 0.2, 0.4, 0.8 or 1.6 mg ml^−1^ from left to right. (c) Groups treated with Ag^+^: 0.723, 1.445, 2.891, 5.781 or 11.560 µg ml^−1^ from left to right.

For assessments of cell viability, a commonly used reagent is TTC, which is a white stain when oxidized. This dye can react with succinate dehydrogenases in living tissues to enter cells and produce the reduced molecule 2,3,5-triphenyltetrazolium formazan (TTF, which appears red (Fig. 1a). We found the culture colours became lighter as the concentration of TiO_2_ (Fig. 1b) or Ag^+^ (Fig. 1c) increased, and sometimes even became colourless. This finding suggested that TiO_2_ and Ag^+^ could inhibit the growth of *S. aureus*. Additionally, this inhibitory effect is increased proportionally with the addition of TiO_2_ and Ag^+^. As changes in colour occurred, the MIC values of TiO_2_ and Ag^+^ against *S. aureus* were 1.6 mg ml^−1^ and 5.781 µg ml^−1^, respectively.

### Effects of TiO_2_ on *S. aureus* growth

**Fig. 2.**
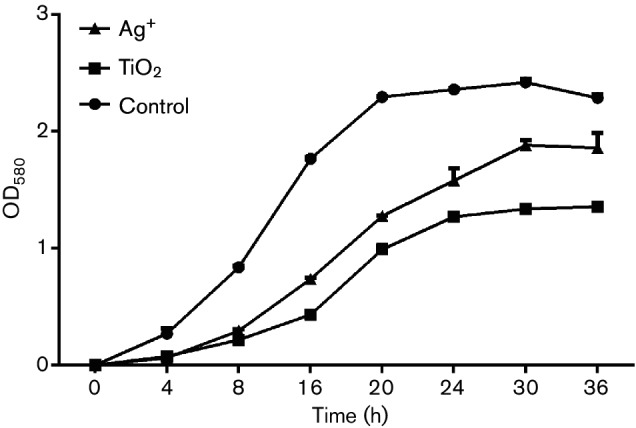
Effects of TiO_2_ and Ag^+^ on *S. aureus* growth curves.

Treatment with TiO_2_ or Ag^+^ could robustly inhibit the growth of *S. aureus*. As shown in Fig. 2, *S. aureus* in the control group rapidly entered log-phase growth after a short lag-phase and reached bacterial stationary phase after an incubation for 24 h. By contrast, the growth rate of *S. aureus* was significantly suppressed after the addition of either TiO_2_ or Ag^+^. The rates of inhibition yielded by either TiO_2_ or Ag^+^ against *S. aureus* were greatest after treatment for 16 h, which were 75.7 % (*P*<0.001) and 58.6 % (*P*<0.001), respectively.

### Effects of TiO_2_ on the surface hydrophobicity of *S. aureus*

**Fig. 3.**
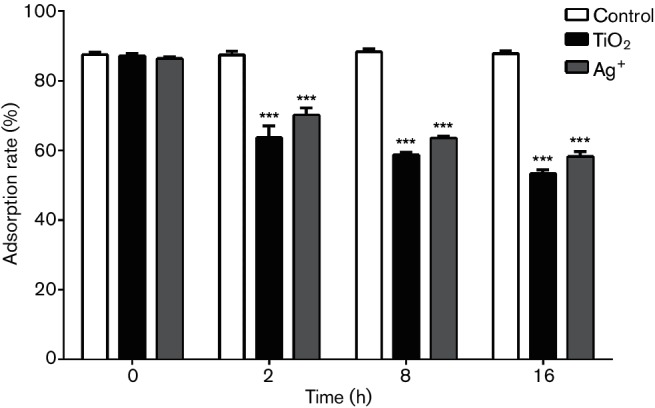
Adsorption rates of *S. aureus* subjected to different treatments (%) (***P<0.001 versus control).

The bacterial cell surface is composed of both hydrophilic and hydrophobic sites, the latter mainly including lipopolysaccharides, lipids and proteins. Bacterial surface hydrophobicity is primarily dependent upon the ratio of proteins and polysaccharides, along with the types and orientations of carbohydrate and polysaccharides in the outer layer of cell. In this present study, we tested effects of TiO_2_ and Ag^+^ on bacterial surface hydrophobicity based on the hydrophobicity of hexadecane and bacterial absorption. As shown in Fig. 3, bacterial surface hydrophobicity was significantly reduced after treatment with either TiO_2_ or Ag^+^. Moreover, the effects of TiO_2_ on *S. aureus* surface hydrophobicity were higher than those of Ag^+^.

### Effects of TiO_2_ on the *S. aureus* membrane

**Fig. 4.**
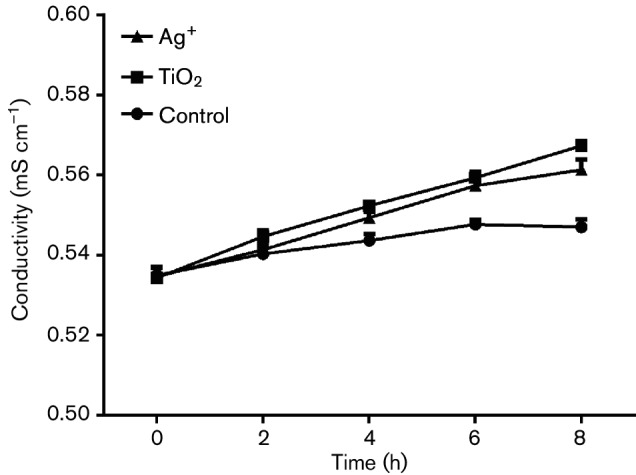
Changes in the conductivity of *S. aureus* in response to TiO_2_ or Ag^+^.

Changes in bacterial membrane permeability can result in the leakage of intracellular small molecules, such as potassium ions and sodium, which can further alter extracellular conductivity [23]. When a bacterial membrane is heavily damaged, DNA and RNA macromolecules will escape from cells. As shown in Fig. 4, the conductivity of cultures increased after treatment with TiO_2_ or Ag^+^.

**Fig. 5.**
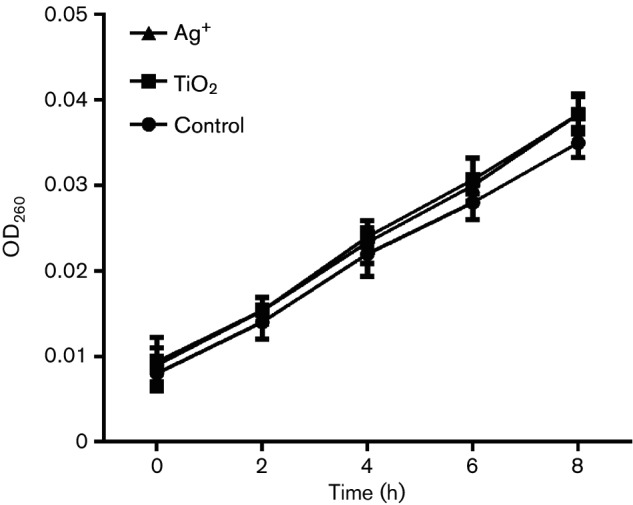
Changes in the OD_260_ of macromolecules from *S. aureus* in response to TiO_2_ or Ag^+^.

Furthermore, we assessed changes in the levels of DNA and RNA macromolecules when cells were treated with TiO_2_ or Ag^+^ to test for effects on membrane permeability in *S. aureus*. As shown in Fig. 5, the content of both DNA and RNA in *S. aureus* showed no obvious changes compared with those observed in the control group.

Together, these findings suggested that treatment with either TiO_2_ or Ag^+^ contributed to changes in membrane permeability in *S. aureus*. This caused slight lesion in the membrane, but not decomposition of membrane, and it did not cause extracellular DNA and RNA leakage.

### Effects of TiO_2_ on the synthesis of soluble proteins in *S. aureus*

**Fig. 6.**
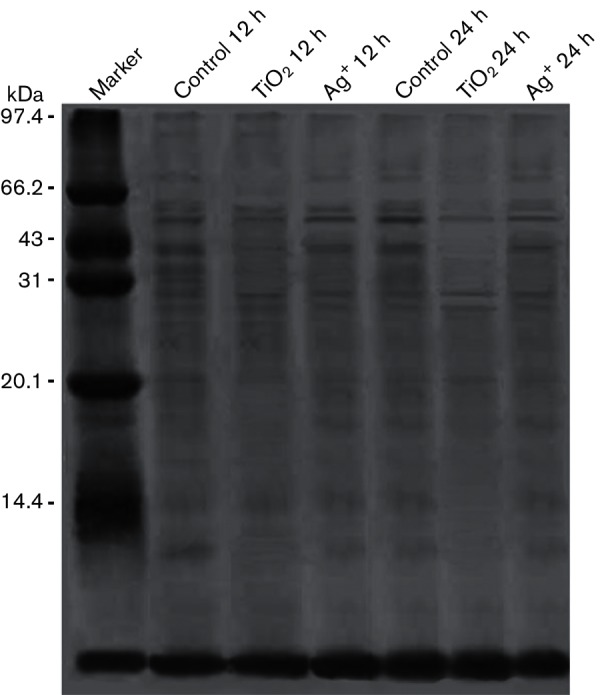
Soluble protein in *S. aureus* treated with TiO_2_ or Ag^+^.

We used SDS-PAGE to determine the soluble protein content in *S. aureus* when cells were treated with TiO_2_ or Ag^+^. As shown in Fig. 6, the addition of TiO_2_ or Ag^+^ to the medium could suppress expression of soluble proteins in *S. aureus*. Moreover, the expression of proteins was further reduced after prolonged treatment with the effectors.

### Effects of TiO_2_ on nucleic acid synthesis in *S. aureus*

**Fig. 7.**
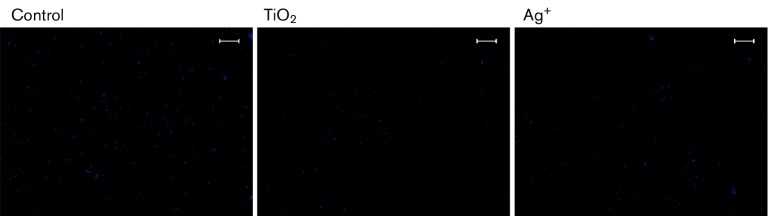
Viability staining of *S. aureus* treated with TiO_2_ or Ag^+^ for 24 h using DAPI (×200). (a) Control. (b) Groups treated with TiO_2_. (c) Groups treated with Ag^+^. Bars, 100 µm.

**Fig. 8.**
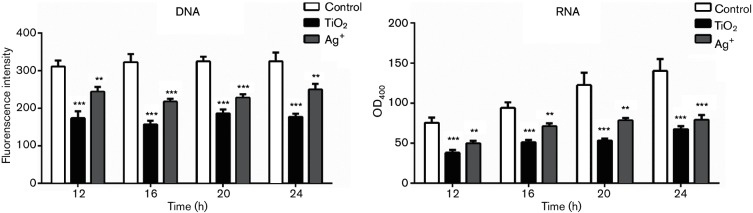
DNA and RNA content of *S. aureus* treated with TiO_2_ or Ag^+^. (a) DNA content of *S. aureus* treated with TiO_2_ or Ag^+^. (b) RNA content of *S. aureus* treated with TiO_2_ or Ag^+^. **P<0.01 versus control; ***P<0.001 versus control.

DAPI is a fluorescent stain that can strongly bind to DNA and RNA, and it is often used to detect nucleic acids. Its fluorescence intensity is positively correlated with the nucleic acid content. In the present study, we studied the effects of TiO_2_ and Ag^+^ on nucleic acid content in *S. aureus* by measuring the fluorescence intensity of cells that had been treated with DAPI. As shown in Fig. 7(a–c), the fluorescence intensity of groups treated with TiO_2_ or Ag^+^ was decreased compared with the control group. Further analysis showed that the fluorescence of both DNA and RNA were significantly decreased in the treated groups (both *P*<0.001), as shown in Tables 1 and 2 and Fig. 8(a, b).

**Table 1.** DNA content of *S. aureus* treated with TiO_2_ and Ag^+^

DNA	12 h	16 h	20 h	24 h
Control	310.67±16.56	322.67±21.55	324.67±12.66	324.67±23.50
TiO_2_	173.67±18.61	156.67±10.02	186.00±10.82	177.00±8.72
Ag^+^	244.00±12.49	217.33±7.64	228.00±9.54	249.67±15.04

**Table 2.** RNA content of *S. aureus* treated with TiO_2_ and Ag^+^

RNA	12 h	16 h	20 h	24 h
Control	75.33±6.51	94.00±7.00	122.33±15.53	140.00±14.93
TiO_2_	38.00±3.61	51.00±3.00	53.00±2.65	67.33±4.04
Ag^+^	49.67±3.06	71.00±3.61	78.33±3.06	79.00±6.24

## DISCUSSION

VAP is a common serious complication in patients with tracheal intubation. The bacteria associated with VAP are almost all antibiotic-resistant strains, such as *S. aureus*, *E. coli* and *Pseudomonas aeruginosa*. TiO_2_ has a strong redox ability and stable chemical properties and bactericidal effect by photocatalysis which can decompose bacteria, viruses and fungi. The sterilizing rate is 99.00 % in no light and 99.99 % in UV light irradiation [24]. Ag^+^, with contact antibacterial action, is the most powerful antibacterial material in inorganic antibacterial materials. In order to effectively control the VAP caused by infection after tracheal intubation, we prepared Ag^+^-loaded TiO_2_ and Ag^+^-loaded SiO_2_ coatings, which could effectively inhibit bacterial growth, such as *S. aureus*, *E. coli* and *P. aeruginosa* [25] and had good biocompatibility [26]. In order to apply TiO_2_ and Ag^+^ in medical instruments, the underling mechanisms of TiO_2_ and Ag^+^ antibacterial effects were explored in the study.

It was found that both TiO_2_ and Ag^+^ could inhibit the growth of *S. aureus*. Moreover, TTC staining analysis indicated that the MIC of TiO_2_ was 1.6 mg ml^−1^, while that of Ag^+^ was 5.781 µg ml^−1^. Therefore, with the same dose, Ag^+^had stronger antibacterial activity than TiO_2_. We used these 0.5 MIC concentrations as working concentrations to explore the mechanisms of TiO_2_ and Ag^+^ against *S. aureus*. Our findings indicated that TiO_2_ and Ag^+^ utilized a similar mechanism against bacterial growth by directly damaging the bacterial surface. The activities of these drugs against negatively charged lipopolysaccharides on the cell surface will result in changes of the surface charge and hydrophobicity, which further affect membrane permeability. However, these changes do not damage intact cells or induce leakage of macromolecules, DNA or RNA. Moreover, treatment with TiO_2_ and Ag^+^ could lead to reduced soluble protein expression by suppressing the synthesis of nucleic acids, which further inhibited the growth of *S. aureus*. The result showed that TiO_2_ and Ag^+^ could cause slight lesion in the membrane to effect *S. aureus* membrane permeability, but not decomposition of membrane, and that TiO_2_ and Ag^+^ could inhibit *S. aureus* nucleic acid synthesis and reduce expression of soluble proteins against bacterial growth. Pleskova *et al*. studied bactericidal activity of TiO_2_ film and also found that *S. aureus* is swollen by TiO_2_ through damaging the cell membrane [27]. An *et al*. also showed that silver nanoparticles could cause DNA to concentrate, lead DNA to lose replication and degrade, and inhibit soluble protein synthesis. Silver nanoparticles could destroy the genetic material of bacteria, so that bacteria could not grow and reproduce [28]. Thus, TiO_2_ and Ag^+^ antibacterial action against *S. aureus* was probably through inhibiting the synthesis of nucleic acid, thereby reducing protein synthesis against bacterial growth.

It should be emphasized that the main aim of this study was to investigate the bactericidal activity of TiO_2_ and Ag^+^ and the underling mechanisms. Although TiO_2_ and Ag^+^ have been shown to exhibit strong bactericidal properties on *S. aureus*, it is important to emphasize that prior to their practical applications in medical instruments, efforts should be made to fully evaluate the potential carcinogenicity and *in vivo* toxicity of TiO_2_ and Ag^+^ against animal cells and tissues.
